# Single Incision versus Conventional Laparoscopic Cholecystectomy Outcomes: A Meta-Analysis of Randomized Controlled Trials

**DOI:** 10.1371/journal.pone.0076530

**Published:** 2013-10-02

**Authors:** Liangyuan Geng, Changhua Sun, Jianfeng Bai

**Affiliations:** Department of General Surgery, the First Affiliated Hospital of Nanjing Medical University, Nanjing, People’s Republic of China; Cardiff University, United Kingdom

## Abstract

**Background:**

Previous meta-analyses that compared the outcome of SILC and CLC have not presented consistent conclusions. This meta-analysis was performed after adding many recent RCTs, to clarify this issue.

**Methods:**

Relevant articles published in English were identified by searching PubMed, Embase, Web of Knowledge, and the Cochrane Controlled Trial Register from January 1997 to February 2013. Reference lists of the retrieved articles were reviewed to identify additional articles. Primary outcomes (postoperative pain scores, cosmetic score, and length of incision) and secondary outcomes (operating time, blood loss, conversion rates, postoperative complications, postoperative hospital stay, time to initial oral intake, and time to resume work) were pooled. Quantitative variables were calculated using the weighted mean difference (WMD), and qualitative variables were pooled using odds ratios (OR).

**Results:**

25 appropriate RCTs were identified from 2128 published articles. 1841 patients were treated, 944 with SILC and 897 with CLC. SILC was superior to CLC in cosmetic score (WMD = 1.155, *P*<0.001), shorter length of incision (WMD = -3.285, *P* = 0.029), and postoperative pain within 12 h (VAS in 3-4 h, WMD = -0.704, *P* = 0.026; VAS in 6-8 h, WMD = -0.613, *P* = 0.010). CLC was superior to SILC in operating time (OT) (WMD = 13.613, *P*<0.001) and need of additional instruments (OR = 7.448, *P*<0.001). Other secondary outcomes were similar.

**Conclusions:**

SILC offered a better cosmetic result and less postoperative pain for patients with uncomplicated cholelithiasis or polypoid lesions of the gallbladder. However, SILC was associated with a longer OT and required additional instruments.

## Introduction

Since the first laparoscopic cholecystectomy (LC) was performed by Mühe et al [1] in 1985, conventional laparoscopic cholecystectomy (CLC) has become the gold standard for treatment of benign gallbladder disease. The concept of minimally invasive surgery has expanded to include smaller wounds and improved cosmesis. Navarra et al [2] first reported transumbilical single-incision laparoscopic cholecystectomy (SILC) in 1997, and proposed that SILC might be associated with less pain and reduced hospitalization. Subsequent comparative studies have reported that SILC was a safe and feasible procedure with better cosmetic results and less postoperative pain [3-5]. There were some drawbacks, however. SILC did not seem to offer any cosmetic advantage over CLC [6], had greater postoperative pain at 4 hours, and was associated with a longer operating time (OT) [7].

Nine meta-analyses [8-16] based on randomized controlled trials (RCTs) have been performed to compare SILC and CLC related outcomes. These studies confirmed the safety and feasibility of SILC. Other findings have not been consistent. Eight meta-analyses showed that SILC offered a better cosmetic score than CLC, while Sajid et al [12] reported no difference between the two. Hao and Arezzo et al [9,16] found that SILC patients have less postoperative pain during the first 24 h, in contrast to the other eight meta-analyses. Two meta-analyses [12,15] noted additional ports had to be inserted with CLC and a higher procedure failure rate with SILC. The other seven meta-analyses did not have these findings. In addition, the number of the RCTs (range: 5-15) included in these meta-analyses was much smaller than more recent RCTs.

These contradictions make it necessary to more closely compare SILC and CLC; in particular, to evaluate whether SILC is associated with less postoperative pain and better cosmetic results and whether SILC is associated with a higher procedure failure rate and longer OTs. This comprehensive meta-analysis included many recent RCTs and was systematically conducted to verify advantages and limits of these two procedures.

## Materials and Methods

A meta-analysis protocol was drafted before the initial search was started. The meta-analysis was conducted and reported according to the Preferred Reporting Items for Systematic Reviews and Meta-Analyses (PRISMA) statement issued in 2009 [17].

### Literature Search

We searched PubMed, Web of Knowledge, Embase and the Cochrane Controlled Trial Register to identify relevant articles published from January 1, 1997 to February 26, 2013 using the search phrases ("single port" OR "single incision" OR "single site" OR “LESS”) AND (laparoscopy OR laparoscopic) AND cholecystectomy. Appropriate adjustments were required according to the database. Filters were used in PubMed, Embase and Web of Knowledge to exclude animal and non-English studies. A manual search of published meta-analyses and relevant articles was performed to identify additional articles.

### Article Selection

The process of article selection was based on the PRISMA [17] flow diagram. Selected studies met the following criteria: (a) RCT design; (b) compared SILC and CLC; (c) revealed at least one of the primary or secondary outcomes mentioned below; and (d) were published in English. Articles were excluded if: (a) the surgery was not cholecystectomy; (b) single incision was not mentioned; (c) it was a retrospective study, prospective nonrandomized study, animal study, review, letter, meeting, or comment. When multiple published articles from the same study were available, the report with the most detailed information was selected.

### Data Extraction

Primary outcomes evaluated included postoperative pain score, cosmetic score, and length of incision. Pain scores from RCTs using a visual analogue scale/score (VAS) were pooled to assess postoperative abdominal pain. Four postoperative time points were used to evaluate pain, 3 to 4 h, 6 to 8 h, 12 h, and 24 h. Cosmetic score was rated using a 10-point scale (0-worst to 10-best).

Secondary measures evaluated included intraoperative outcomes (OT, blood loss, conversion rates), postoperative complications (wound complications, incisional hernia, bile leakage, retained stones, bile duct injury and bleeding), and recovery outcomes (length of postoperative hospital stay, time to initial oral intake, and time to resume work). Conversion rates included operations that were converted to open or that required additional instruments. Additional added instruments were defined as situations where it was necessary to use more trocars than planned or where they were needed to enhance the exposure of Calot’s triangle for gallbladder retraction.

Patient characteristics (number of patients, gender, age, body mass index, American Society of Anesthesiology rating, presence of acute cholecystitis, history of prior gastrointestinal surgery, surgical technique, and follow-up time) were also recorded. If the above data was not available in the published study, the authors were contacted and asked to supply the information.

### Assessment of Study Quality

The literature search, article selection, data extraction and assessment of study quality were completed independently by two authors (Geng and Sun). Discrepancies were resolved by discussion. When a consensus could not be reached, a third author (Bai) broke the tie. The Jadad’s revised rating scale [18] of each RCT is shown in Table 1. RCT randomization that was performed using a computer generated number and concealed in an opaque and sealed envelope (or similar method) was considered appropriate. Use of a non-transparent dressing covering the abdomen (or similar method) during the entire hospitalization was considered to be appropriate double blinding. A scale from 4 to 7 was considered a high-quality RCT.

**Table 1:** Jadad’s revised rating scales of the 25 studies included in the meta-analysis.

		**Randomization**				
**Study**	**Country**	**Generate**	**Hide**	**Double Blinding**	**Withdraws and Dropouts**	**Jadad**’s **Score** ^a^
Saad [22], 2013	Germany	2	2	2	1	7
Madureira [23], 2013	Brazil	1	0	0	1	2
Chang [24], 2013	Singapore	1	0	1	1	3
Ostlie [25], 2013	USA	2	0	0	0	2
Pan [26], 2013	China	2	2	0	1	5
Sinan [27], 2012	Turkey	2	1	0	0	3
Vilallonga [28], 2012	Spain	1	0	0	0	1
Phillips [29], 2012	USA	1	0	2	1	4
Noguera [30], 2012	Spain	1	0	0	0	1
Sasaki [31], 2012	Japan	2	0	0	1	3
Luna [32], 2012	Brazil	1	0	0	0	1
Leung [33], 2012	USA	1	0	2	0	3
Zheng [34], 2012	China	2	2	0	0	4
Marks [35], 2011	USA	1	1	1	0	3
Ma [36], 2011	Portland	2	1	0	0	3
Lirici [37], 2011	Italy	2	2	2	0	6
Lai [38], 2011	China	2	2	0	1	5
Cao [39], 2011	China	1	2	0	0	3
Bucher [40], 2011	Switzerland	2	1	0	1	4
Aprea [41], 2011	Italy	1	2	0	0	3
Tsimoyiannis [42], 2010	Greece	1	2	1	0	4
Lee [43], 2010	China	1	2	0	1	4
Mehamood [44], 2010	Pakistan	1	2	0	0	3
Rasic [45], 2010	Croatia	2	0	0	0	2
Bresadola [46], 1999	Italy	1	0	0	0	1

^a^ 4 to 7 as high-quality, otherwise, considered low-quality.

### Statistical Analysis

Continuous variables (OT, blood loss, postoperative hospital stay, time to initial oral intake, time to resume work, postoperative pain score, cosmetic score, and length of incision) were combined using the weighted mean difference (WMD). The method of Hozo et al [19] was used if variables were provided as medians or/and ranges instead of a mean with a standard deviation. Binary variables (conversion rate, postoperative complications) were pooled using an odds ratio (OR).

Homogeneous data was evaluated using fixed effect models. The inverted variance method was used for continuous variables and the Mantel-Haenszel method for binary variables. Random effect models based on the DerSimonian & Laird method were used to calculate the combined outcomes of both continuous and binary variables when heterogeneity existed. *P*<0.05 was considered statistically significant.

Heterogeneity was identified using a chi-square-based Q-test (*P*≤0.10) and *I* ^2^ index (*I* ^2^ exceeding 50 percent). If heterogeneity was found, a meta-regression based on the Restricted Maximum Likelihood (REML) method was conducted to identify any related factors (*P*<0.05 was considered significant). Subgroup analyses were conducted to identify potential sources of heterogeneity when the meta-regression was not adequate (less than 10 studies reported the outcome) or as a supplementary method.

Sensitivity analyses were performed to examine the effect of excluding lower quality studies. Publication bias was evaluated using Egger’s regression test, with *P*<0.05 indicating statistically significant publication bias. The confidence interval (CI) was established at 95%. Statistical analyses were carried out using Stata11.0 software (Stata Corporation, USA).

## Results

### Identification of Studies and Quality of the RCTs

**Figure 1:**
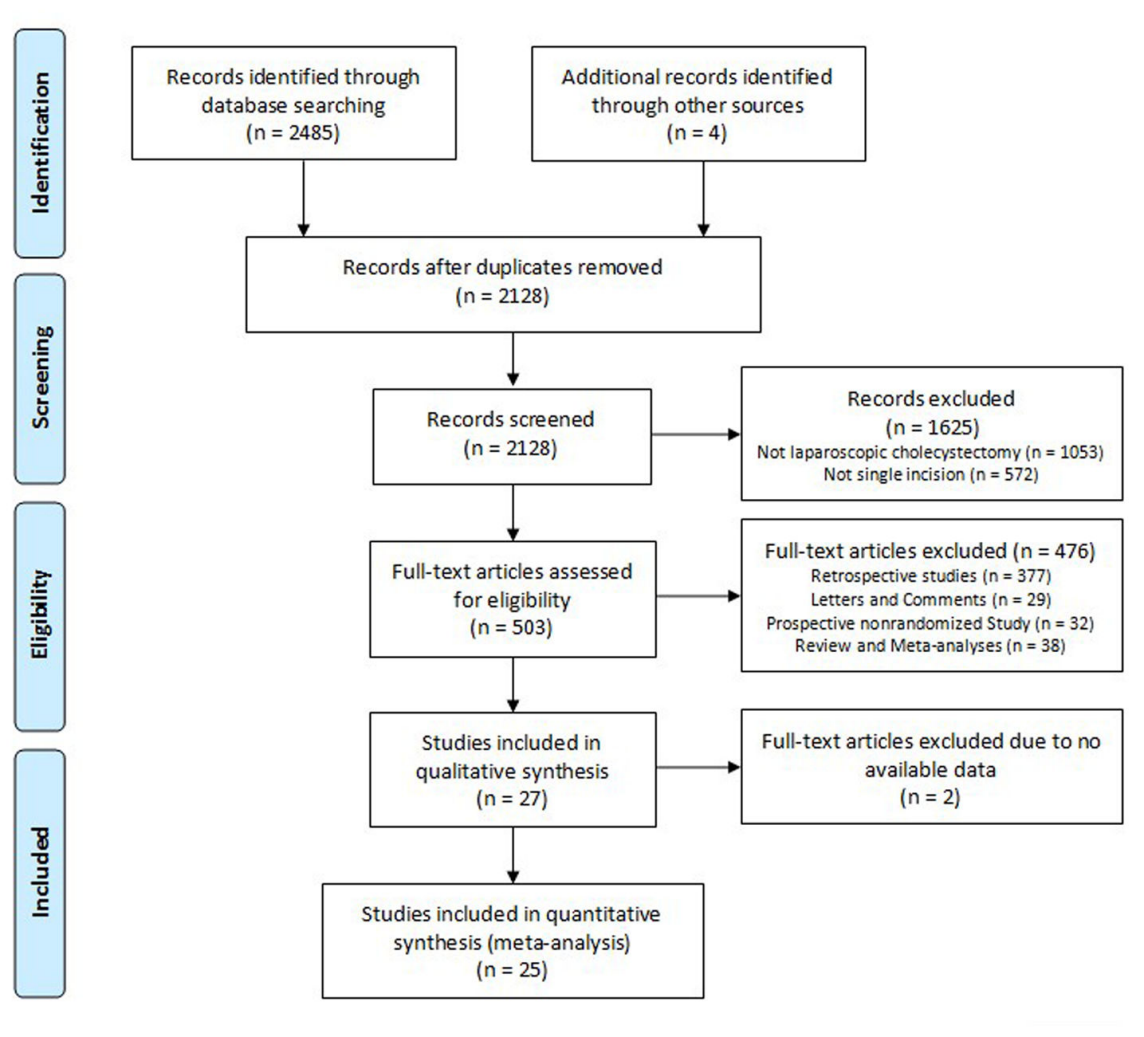
PRISMA flow diagram of the systematic article selection process.

27 RCTs [20-46] were extracted from 2128 publications identified from databases and other sources. Requested data could not be obtained from the author in two ongoing trials [20,21]. The PRISMA [17] flow diagram for this meta-analysis is presented in Figure 1. Many studies were called randomized without defining the method of randomization or blinding and whether patients withdrew or dropped out (Table 1). Only nine high-quality (Jadad’s revised rating scale, 4 to 7) articles were included in the meta-analysis (Table 1).

### Characteristics of Included RCTs

1841 patients (944 with SILC, 897 with CLC) were identified to be included in the meta-analysis. Table S1 shows the general characteristics, including sample size, M/F ratio, age, body mass index (BMI), and American Society of Anesthesiology (ASA) score. Patients with a history of gastrointestinal surgery were reported by Ma (67% in SILC, and 76% in CLC) and Bucher (39% in SILC, and 36% in CLC) et al [36,40]. Only Vilallonga, Leung and Bucher et al [28,33,40] reported SILC in patients with acute cholecystitis. CLC was performed using 3 or 4 ports. Four techniques were used to perform SILC. Follow-up in the RCTs was usually short. General characteristics, operative techniques and follow-up time of the 25 studies are listed in Tables S1 and S2.

### Quantitative Synthesis

#### Primary Outcomes

**Figure 2:**
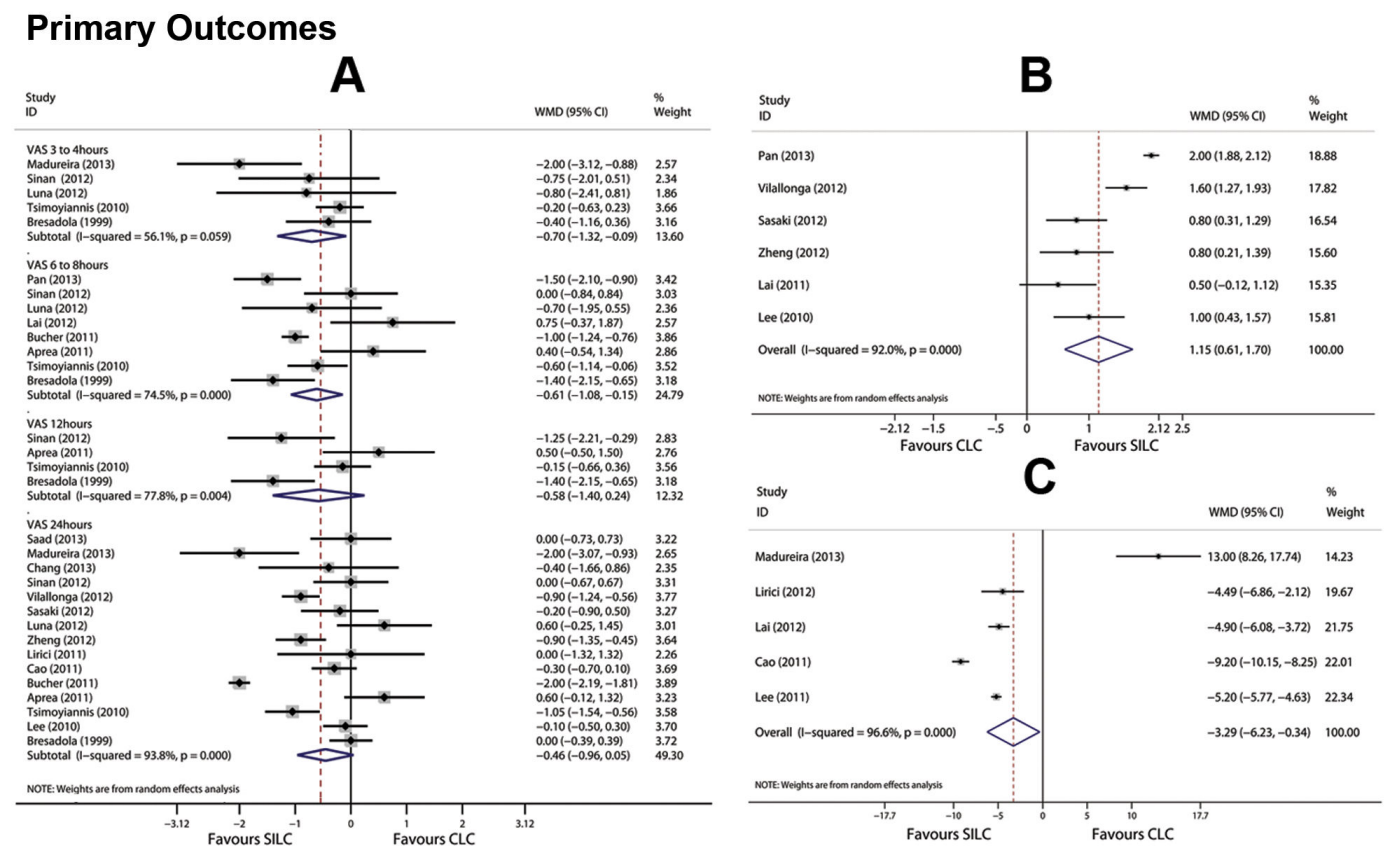
Forest plots for primary outcomes included postoperative pain scores from four time points(A) cosmetic score (B) and length of incision (C). CI: confidence interval; WMD: weighted mean difference. Random effects models based on the DerSimonian & Laird methods were used as heterogeneity existed in all primary outcomes.

The postoperative pain scores were assessed in twenty one studies (Table S3). Table 2 summarizes the pooled results of the VASs. VASs from postoperative 3 to 4 h and 6 to 8h were significantly lower after SILC (*P* = 0.026 and 0.010, respectively). There were no significant differences in VASs at 12 h and 24h (*P* = 0.168 and 0.076, respectively). Thirteen studies reported cosmetic score. Three were excluded as they did not provide a ten-point scale (Table S4). The best cosmetic result was obtained with SILC (*P*<0.001) (Table 2). Five studies reported the length of the incision, and SILC was compared with three-port LC in one article (Table S4). The length of incision for SILC was significantly shorter than that with CLC (*P* = 0.029) (Table 2). Forest plots of primary outcomes are listed in Figure 2.

**Table 2:** Meta-analysis of the primary and secondary outcomes in 25 RCTs.

		**Quantitative Synthesis**				**Heterogeneity**		**Bias**
**Outcomes**		**WMD/OR**	**95% CI**	***z***	***P***	***I^2^***	***P***	***P***
**Primary Outcomes**								
Postoperative Pain Score^a^								
	VAS in 3 to 4h	-0.704	-1.323, -0.085	2.23	0.026	56.1%	0.059	0.184
	VAS in 6 to 8h	-0.613	-1.077, -0.149	2.59	0.010	74.5%	< 0.001	0.174
	VAS in 12h	-0.580	-1.404, 0.244	1.38	0.168	77.8%	0.004	0.763
	VAS in 24h	-0.457	-0.963, 0.048	1.77	0.076	93.8%	< 0.001	0.007
Cosmetic Score^a^		1.155	0.607, 1.703	4.13	< 0.001	92.0%	< 0.001	0.001
Length of Incision^a^		-3.285	-6.232, -0.338	2.18	0.029	96.6%	< 0.001	0.451
**Secondary Outcomes**								
Intraoperative Outcomes								
	Operating Time^a^	13.613	9.047, 18.179	5.84	< 0.001	90.1%	< 0.001	0.005
	Blood Loss^a^	1.506	-1.666, 4.679	0.93	0.352	72.0%	0.001	0.889
	Additional Instrument Added	7.448	3.821, 14.518	5.90	< 0.001	0	0.867	0.931
	Conversion to Open	0.686	0.132, 3.576	0.45	0.655	0	0.575	0.724
Postoperative Complications								
	Wound Complications	1.336	0.842, 2.119	1.23	0.219	0	0.556	0.393
	Incisional Hernia	1.937	0.658, 5.706	1.20	0.230	0	0.907	0.422
	Bile Leakage	1.329	0.451, 3.912	0.52	0.606	0	0.631	0.878
	Retained Stones	2.149	0.554, 8.329	1.11	0.269	0	0.933	< 0.001
	Bile Duct Injury	1.000	0.165, 6.066	0.70	1.000	0	0.364	-
	Bleeding	0.586	0.074, 4.639	0.51	0.613	0	0.602	-
	Overall Morbidity	1.220	0.888, 1.676	1.23	0.220	0	0.903	0.689
Recovery Outcomes								
	Postoperative Hospital Stay^a^	-0.127	-0.384, 0.129	0.74	0.331	91.8%	< 0.001	0.680
	Initial Oral Intake	-0.196	-1.204, 0.813	0.38	0.704	0	0.775	0.012
	Time to Resume Work^a^	-0.527	-2.122, -1.068	0.65	0.517	94.5%	< 0.001	0.734

Continuous variables were combined by weighted mean difference (WMD); binary variables were pooled by odds ratios (OR). a: Random effect model was used; otherwise, fixed-effects model was used.

#### Secondary Outcomes

**Figure 3:**
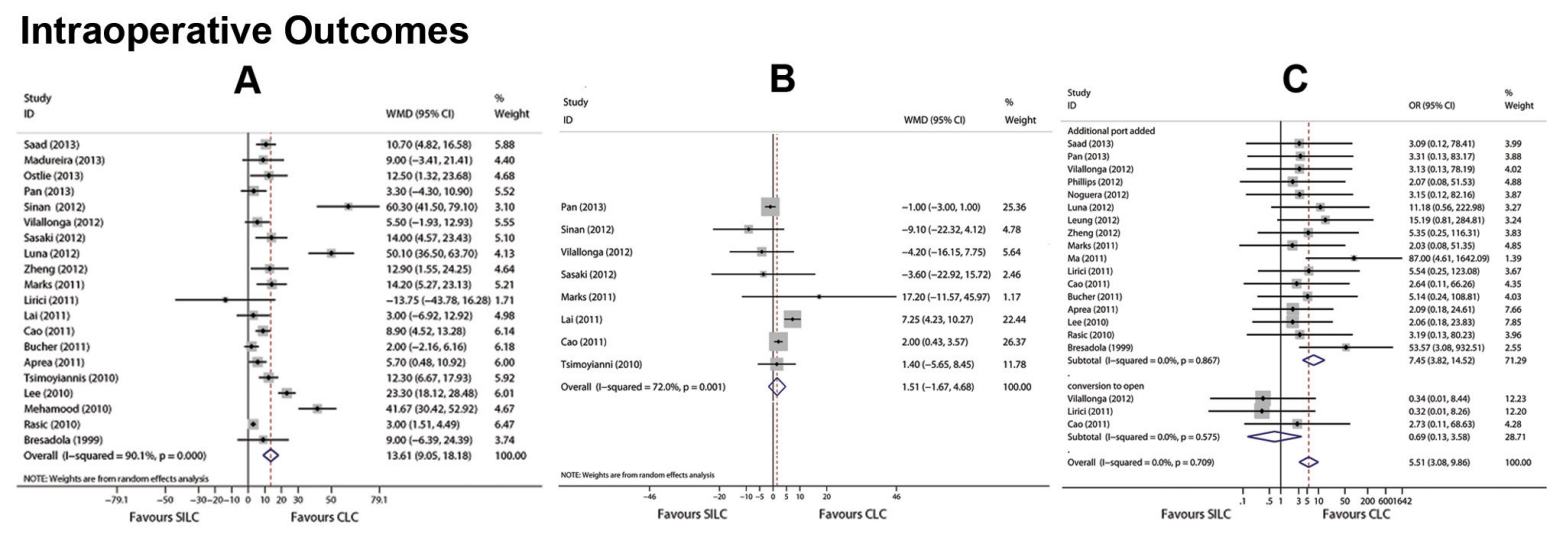
Forest plots for intraoperative outcomes included operating time (A) **blood loss (B) conversion rate (C)**. CI: confidence interval; WMD: weighted mean difference; OR: odds ratio. A fixed effect model was used as no statistical heterogeneity across conversion rate (C) was observed. Random effects models were used as heterogeneity existed in operating time (A) and blood loss (B).

25 studies reported OT. Five studies were excluded because there was no standard deviation (Table S5). OT with SILC was significantly longer than that with CLC (*P*<0.001). There was no difference in blood loss in 9 reporting articles (*P* = 0.352). Pooled results of OT and blood loss are listed in Table 2. The incidence of additional instruments used for SILC and CLC was 6.1% and 0.2%, respectively (Table S5). It was statistically higher for SILC than that with CLC (*P*<0.001) (Table 2). Among the three patients converted to open surgery, one occurred with SILC and two occurred with CLC (Table S5). The conversion to open rates of SILC and CLC were 0.1% and 0.2%, respectively. There was no significant difference in the conversion to open rate (*P* = 0.655) (Table 2). Forest graphs for intraoperative outcomes are shown in Figure 3.

**Figure 4:**
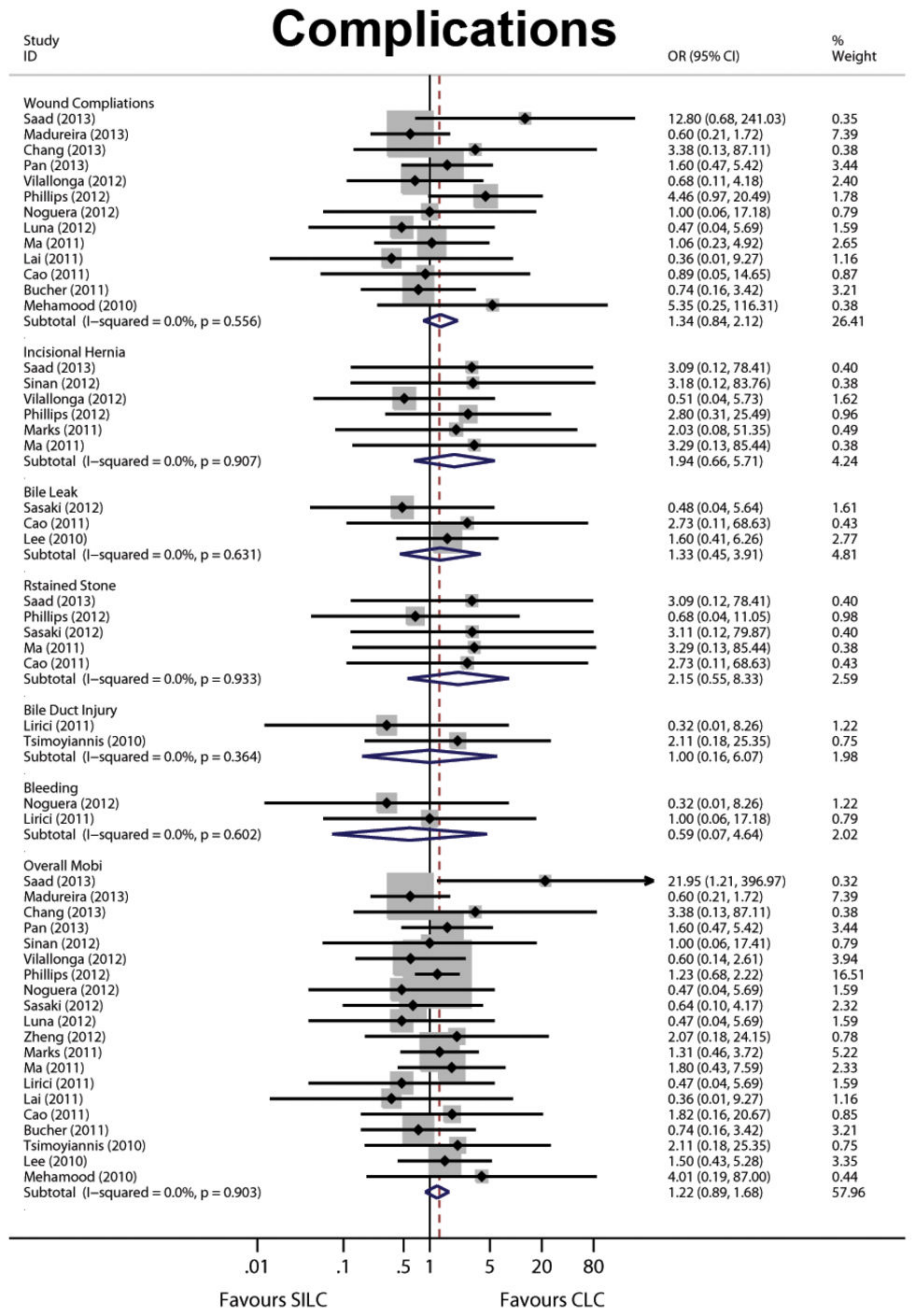
Forest plots for postoperative complications included wound complications, incisional hernia, bile leak, retained stones, bile duct injury and bleeding. CI: confidence interval; OR: odds ratio. A fixed effects model was used as no statistical heterogeneity across complications were observed.

Only three articles included in the meta-analysis showed no complication (Table S6). The overall morbidity was 12.39% (117) for SILC and 9.48% (85) for CLC (*P* = 0.220) (Table 2). There were also no differences in the wound complications, incisional hernia, bile leakage, retained stones, bile duct injury and bleeding sub-groups (*P*>0.05) (Table 2). There were no deaths in any of the included RCTs. Forest graphs for postoperative complications are shown in Figure 4.

**Figure 5:**
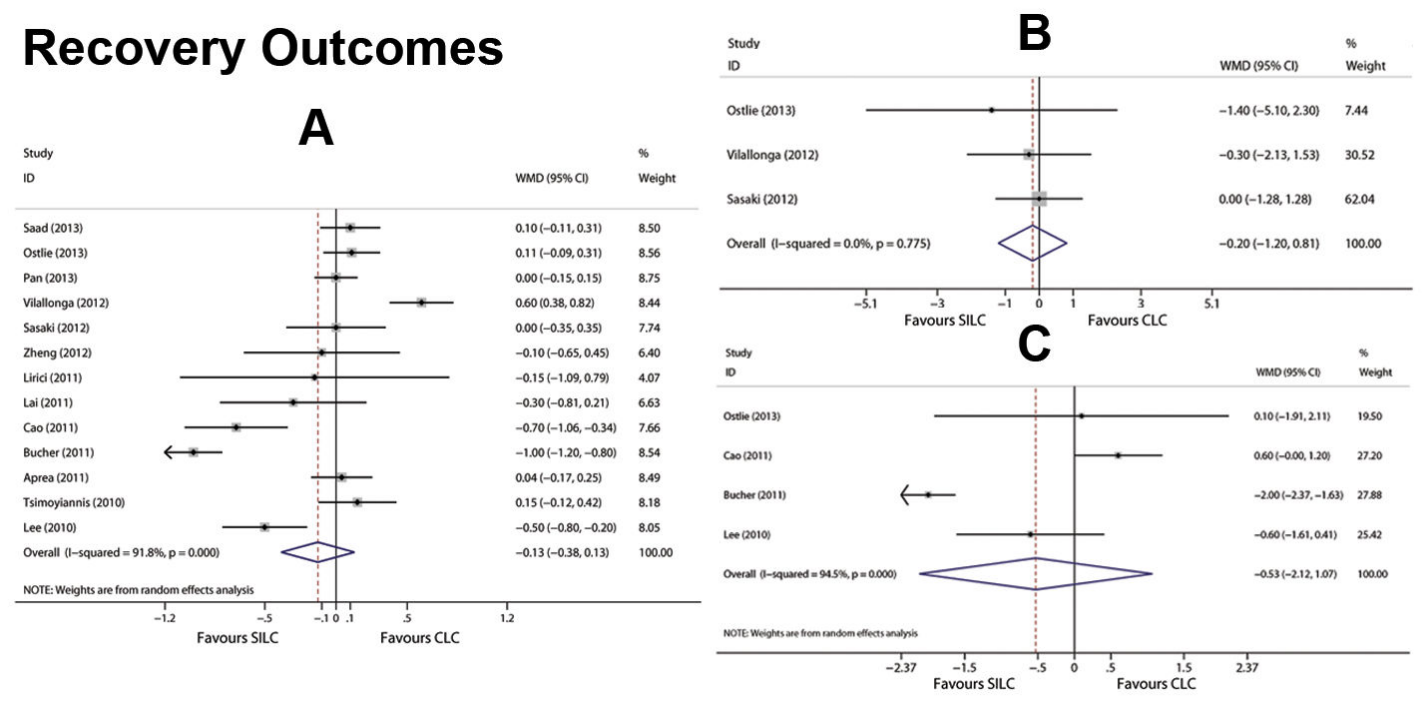
Forest plots for recovery outcomes included length of postoperative hospital stay (A), time to initial oral intake (B) and time to resume work (C). CI: confidence interval; WMD: weighted mean difference. Random effects models based on the DerSimonian & Laird methods were used as heterogeneity existed in hospital stay (A) and time to resume work (C). A fixed effect model was used as no statistical heterogeneity across initial oral intake (B) was observed.

Pooled results of recovery outcomes are listed in Table 2. There was no significant difference in the postoperative hospital stay, time to initial oral intake, and time to resume work of patients undergoing SILC and CLC in 17 articles (*P*>0.05). The raw data of recovery outcomes are listed in Table S7. Forest graphs for recovery outcomes are shown in Figure 5.

### Test of Heterogeneity

The results of heterogeneity testing are summarized in Table 2. Significant heterogeneity existed in all primary outcomes (postoperative pain scores, cosmetic score and length of incision) and some secondary outcomes (OT, blood loss, postoperative hospital stay and time to resume work).

We evaluated study quality and general and secondary characteristics of the included studies (Table 1, S1 and S2) as potential sources of methodological and clinical heterogeneity. Meta-regressions were performed for postoperative pain scores, OT and hospital stay to assess the potential reasons. Subgroup analyses were conducted by stratifying study quality (high-quality vs. low-quality, Table 1), acute cholecystitis (SILC patients with vs. without), operative technique and follow-up time (short vs. slight long period) (Table S2) to verify the accuracy of the meta-regression and assess the possible sources of heterogeneity in cosmetic score, length of incision, blood loss and time to resume work. There were no significance sources of methodological and clinical heterogeneity identified by the meta-regression and subgroup analyses (Data not shown).

### Sensitivity Analysis and Publication Bias

Sensitivity analysis was conducted to assess the effect of study quality. Only length of incision results were affected by (low) quality of study (Data not shown). After low-quality studies were excluded, there was no statistically difference in length of incision (*P* = 0.841). Statistical publication bias was found in VAS in 24h, cosmetic score, OT, retained stones, and time to initial oral intake, according to Egger’s test (*P*<0.05) (Table 2).

## Discussion

A better cosmetic score, length of incision, and less postoperative pain within 12 h were found with SILC. CLC was associated with a shorter OT and required fewer additional instruments. There was no significant difference between SILC and CLC in regard to blood loss, open conversion rate, postoperative complications, time of hospital stay, time to initial oral intake, and time to resume work.

There were several limitations to this study. The meta-regression and subgroup analyses we performed did not account for all the sources of heterogeneity, which existed in the great majority of continuous variables (Table 2). Random effects models were used when heterogeneity existed, although the stability of the pooled analyses could not be affirmed. There was also publication bias in some of the outcomes. One potential reason is that there were many non-double-blind studies with only a small number of cases enrolled. No withdrawals or dropouts were reported in the majority of articles, and Jadad’s revised rating scale for the RCTs was low (Table 1). In addition, complicated cases such as acute cholecystitis were excluded from most of studies except that of Vilallonga, Leung and Bucher et al [28,33,40]. We attempted to avoid sampling bias by requesting missing data from all the RCTs. We were not able to obtain this data from two reports [20,21]. Finally, we performed an electronic search and a manual search in order to identify any potentially relevant articles. We may have missed some meaningful articles, especially those not in English.

A major focus of this study was to determine which surgical method was associated with the least postoperative pain and best cosmetic results. In contrast to previous meta-analyses, we found a significant difference in the VAS scores at postoperative 3 to 4h and 6 to 8h. Patients undergoing SILC may have had less pain due to absence of the subcostal and xiphoid incision. The postoperative VASs could be influenced by bile leakage, intraperitoneal pressure, use of local anesthetics, peritoneal irrigation, psychological factors and type of incision [38,47-49]. These factors could also contribute to heterogeneity. Although all of RCTs reported a postoperative pain score, different time points and methods were used (Table S3). The presence of heterogeneity and publication bias prevented identification of a superior surgical technique. Future prospective double-blind randomized controlled studies will need to address the issue of postoperative pain at different time points.

Most reports documented an improved cosmetic result after SILC [40,43,50]. SILC produced a shorter incision with a smaller scar. An important reason for the use of SILC was that scarless surgery is a high priority. Our study demonstrated that SILC was associated with a better cosmetic score and a shorter incision than CLC. Our findings regarding cosmetic score were identical to seven previous meta-analyses. The better cosmetic results with SILC may be explained by the use of a single concealed umbilical incision of short length. It was difficult to evaluate cosmetic score because of the subjective factors and environmental impact [36]. The scar may change over time, thus resulting in different outcomes. Also, the evaluation of cosmetic results currently only evaluates the postoperative abdominal incision. We found an unstable result regarding length of incision after low-quality RCTs were excluded. Large high-quality RCTs are needed to assess cosmetic results.

SILC was associated with a significantly longer OT than CLC, similar to all the other meta-analyses except that of Zhong et al [14]. It has been observed that the OT of SILC decreased significantly after the initial 10 cases, as experience is gained. No significance difference in the OT of SILC and CLC was reported after the first 10 cases [5,46]. Only the surgeons performing the operations in one RCT included in our meta-analysis were beyond the SILC learning curve phase with proficient experience [40]. This could be partly responsible for a long OT in the SILC group and may explain the heterogeneity and publication bias. In addition, Cao et al [39] found that SILC patients with a high BMI (>24), acute cholecystitis, or a history of abdominal surgery with related abdominal adhesions exhibited longer OTs than similar CLC patients. Zahid et al [51] also reported that the inflammation and adhesions of the gallbladder, and BMI were important factors affecting OT. These types of patients were rarely included in RCTs and could explain the publication bias. A large RCT including complicated cases need to be performed. Surgeons in the study should be experienced and beyond their learning curve.

We found SILC was associated with an increased requirement for additional port insertion or instruments, similar to the meta-analysis by Sajid et al [12]. Ma et al [36] reported that 78.6 percent (11/14) additional instruments added cases in SILC were present in their learning curve phase. Little experience in SILC initial attempt could be responsible for the need to put additional ports or instruments. Furthermore, SILC could be converted to CLC in patients with acute cholecystitis, dense adhesions, Mrizzi syndrome and obesity, where there were technical problems and difficulties identifying anatomic landmarks [32,34,39,40,46].

SILC and CLC had a similar rate of postoperative complications. Wound infections were a major concern after SILC. The umbilical port could be seeded during removal of the gallbladder or with inadequate port site inspection or closure [29,52]. While SILC may be more prone to incisional hernias than CLC, because of the longer incision, we found no difference in the two methods. Follow-up was short, however, and may underestimate the final results. Hall et al [52] reported that transperitoneal sutures might increase the risk of intraoperative bile leakage. This meta-analysis found no difference in bile leakage in the two methods. Only Bresadola and Aprea et al [41,46] performed intraoperative cholangiography, which could lead to a publication bias, as it may reduce the rate of retained stones. The rate of bile duct injury was similar in both groups. This was expected as most patients were not technically complicated.

While there have been some reports of a benefit in hospital stay with SILC [40,43,50], we found no such benefit. This finding was supported by the majority of previous meta-analyses. There was no significant difference in time to resume work and time to initial oral intake. We believe that the small incision used for SILC was not significantly different from that in CLC, and did not affect recovery. Many other factors affected measurement of recovery including hospital factors, social habits, and medical insurance and so on [53]. These factors could contribute to heterogeneity and publication bias.

## Conclusions

SILC was the preferred procedure for the treatment of uncomplicated gallbladder stones and polyps, as it was associated with a better cosmetic result and less postoperative pain. There was not enough data to support SILC as the standard of care as it was associated with longer OTs and more frequently required additional instruments. A large prospective double-blind randomized controlled trial comparing SILC and CLC is needed to identify the best procedure. The presence of a learning curve for the surgeons needs to be accounted for. The effect of complicating patient factors, including acute cholecystitis, previous abdominal surgery, and severe obesity, need to identified.

## Supporting Information

Checklist S1 **PRISMA 2009 Checklist.**
(DOC)

Table S1 **General characteristics of the 25 studies included in the meta-analysis.**
(DOC)

Table S2 **Operative techniques and follow-up time in the 25 studies.**
(DOC)

Table S3 **VASs of the 25 studies included in the meta-analysis.**
(DOC)

Table S4 **Cosmetic results of the 25 studies included in the meta-analysis.**
(DOC)

Table S5 **Intraoperative outcomes of the 25 studies included in the meta-analysis.**
(DOC)

Table S6 **Postoperative complications of the 25 studies included in the meta-analysis.**
(DOC)

Table S7 **Recovery outcomes of the 25 studies included in the meta-analysis.**
(DOC)

## References

[1] Mühe E (1991) Laparoscopic cholecystectomy--late results. Langenbecks Arch Chir Suppl Kongressbd: 416-423. PubMed: 1838946. 1838946 10.1007/978-3-642-95662-1_189

[2] Navarra G, Pozza E, Occhionorelli S, Carcoforo P, Donini I (1997) One-wound laparoscopic cholecystectomy. Br J Surg 84: 695. doi:10.1002/bjs.1800840536. PubMed: 9171771. 9171771

[3] Joseph S, Moore BT, Sorensen GB, Earley JW, Tang F et al. (2011) Single-incision laparoscopic cholecystectomy: a comparison with the gold standard. Surg Endosc 25: 3008-3015. doi:10.1007/s00464-011-1661-x. PubMed: 21487878. 21487878 10.1007/s00464-011-1661-x

[4] Hauters P, Auvray S, Cardin JL, Papillon M, Delaby J et al. (2013) Comparison between single-incision and conventional laparoscopic cholecystectomy: a prospective trial of the Club Coelio. Surg Endosc 27: 1689-1694. doi:10.1007/s00464-012-2657-x. PubMed: 23224032. 23224032 10.1007/s00464-012-2657-x

[5] Solomon D, Shariff AH, Silasi DA, Duffy AJ, Bell RL et al. (2012) Transvaginal cholecystectomy versus single-incision laparoscopic cholecystectomy versus four-port laparoscopic cholecystectomy: a prospective cohort study. Surg Endosc 26: 2823-2827. doi:10.1007/s00464-012-2253-0. PubMed: 22549370. 22549370 10.1007/s00464-012-2253-0

[6] Garg P, Thakur JD, Raina NC, Mittal G, Garg M et al. (2012) Comparison of cosmetic outcome between single-incision laparoscopic cholecystectomy and conventional laparoscopic cholecystectomy: an objective study. J Laparoendosc Adv Surg Tech A 22: 127-130. doi:10.1089/lap.2011.0391. PubMed: 22145988. 22145988 10.1089/lap.2011.0391

[7] Han HJ, Choi SB, Kim WB, Lee JS, Boo YJ et al. (2012) Surgical stress response and clinical outcomes of single port laparoscopic cholecystectomy: prospective nonrandomized study. Am Surg 78: 485-491. PubMed: 22472410. 22472410

[8] Garg P, Thakur JD, Garg M, Menon GR (2012) Single-incision laparoscopic cholecystectomy vs. conventional laparoscopic cholecystectomy: a meta-analysis of randomized controlled trials. J Gastrointest Surg 16: 1618-1628. doi:10.1007/s11605-012-1906-6. PubMed: 22580841. 22580841 10.1007/s11605-012-1906-6

[9] Hao L, Liu M, Zhu H, Li Z (2012) Single-incision versus conventional laparoscopic cholecystectomy in patients with uncomplicated gallbladder disease: a meta-analysis. Surg Laparosc Endosc Percutan Tech 22: 487-497. doi:10.1097/SLE.0b013e3182685d0a. PubMed: 23238374. 23238374 10.1097/SLE.0b013e3182685d0a

[10] Markar SR, Karthikesalingam A, Thrumurthy S, Muirhead L, Kinross J et al. (2012) Single-incision laparoscopic surgery (SILS) vs. conventional multiport cholecystectomy: systematic review and meta-analysis. Surg Endosc 26: 1205-1213. doi:10.1007/s00464-011-2051-0. PubMed: 22173546. 22173546 10.1007/s00464-011-2051-0

[11] Pisanu A, Reccia I, Porceddu G, Uccheddu A (2012) Meta-analysis of prospective randomized studies comparing single-incision laparoscopic cholecystectomy (SILC) and conventional multiport laparoscopic cholecystectomy (CMLC). J Gastrointest Surg 16: 1790-1801. doi:10.1007/s11605-012-1956-9. PubMed: 22767084. 22767084 10.1007/s11605-012-1956-9

[12] Sajid MS, Ladwa N, Kalra L, Hutson KK, Singh KK et al. (2012) Single-Incision laparoscopic cholecystectomy versus conventional laparoscopic cholecystectomy: meta-analysis and systematic review of randomized controlled trials. World J Surg 36: 2644-2653. doi:10.1007/s00268-012-1719-5. PubMed: 22855214. 22855214 10.1007/s00268-012-1719-5

[13] Wang Z, Huang X, Zheng Q (2012) Single-incision versus conventional laparoscopic cholecystectomy: a meta-analysis. ANZ J Surg 82: 885-889. doi:10.1111/j.1445-2197.2012.06284.x. PubMed: 23009184. 23009184 10.1111/j.1445-2197.2012.06284.x

[14] Zhong X, Rui YY, Zhou ZG (2012) Laparoendoscopic single-Site versus traditional laparoscopic surgery in patients with cholecystectomy: a meta-analysis. J Laparoendosc Adv Surg Tech A 22: 449-455. doi:10.1089/lap.2011.0521. PubMed: 22670637. 22670637 10.1089/lap.2011.0521

[15] Trastulli S, Cirocchi R, Desiderio J, Guarino S, Santoro A et al. (2013) Systematic review and meta-analysis of randomized clinical trials comparing single-incision versus conventional laparoscopic cholecystectomy. Br J Surg 100: 191-208. doi:10.1002/bjs.8937. PubMed: 23161281. 23161281 10.1002/bjs.8937

[16] Arezzo A, Scozzari G, Famiglietti F, Passera R, Morino M (2013) Is single-incision laparoscopic cholecystectomy safe? Results of a systematic review and meta-analysis. Surg Endosc 27: 2293-2304. doi:10.1007/s00464-012-2763-9. PubMed: 23355161. 23355161 10.1007/s00464-012-2763-9

[17] Moher D, Liberati A, Tetzlaff J, Altman DG, Grp P (2009) Preferred Reporting Items for Systematic Reviews and Meta-Analyses: The PRISMA Statement. Plos Medicine 6

[18] Jadad AR, Moore RA, Carroll D, Jenkinson C, Reynolds DJ et al. (1996) Assessing the quality of reports of randomized clinical trials: is blinding necessary? Control Clin Trials 17: 1-12. doi:10.1016/S0197-2456(96)90740-0. PubMed: 8721797. 8721797 10.1016/0197-2456(95)00134-4

[19] Hozo SP, Djulbegovic B, Hozo I (2005) Estimating the mean and variance from the median, range, and the size of a sample. BMC Med Res Methodol 5: 13. doi:10.1186/1471-2288-5-13. PubMed: 15840177. 15840177 10.1186/1471-2288-5-13 PMC1097734

[20] Steinemann DC, Raptis DA, Lurje G, Oberkofler CE, Wyss R et al. (2011) Cosmesis and body image after single-port laparoscopic or conventional laparoscopic cholecystectomy: a multicenter double blinded randomised controlled trial (SPOCC-trial). BMC Surg 11: 24. doi:10.1186/1471-2482-11-24. PubMed: 21910897. 21910897 10.1186/1471-2482-11-24 PMC3189390

[21] Tacchino R, Marks J, Onders R, DeNoto G, Paraskeva B et al. (2011) Conventional 4-port laparoscopic cholecystectomy versus silstm port laparoscopic cholecystectomy. The first prospective randomized sham controlled trial. Surg Endosc 25: S35. 10.1007/s00464-011-2028-z 22083331

[22] Saad S, Strassel V, Sauerland S (2013) Randomized clinical trial of single-port, minilaparoscopic and conventional laparoscopic cholecystectomy. Br J Surg 100: 339-349. doi:10.1002/bjs.9003. PubMed: 23188563. 23188563 10.1002/bjs.9003

[23] Madureira FA, Manso JE, Madureira Fo D, Iglesias AC (2013) Randomized clinical study for assessment of incision characteristics and pain associated with LESS versus laparoscopic cholecystectomy. Surg Endosc 27: 1009-1015. doi:10.1007/s00464-012-2556-1. PubMed: 23052531. 23052531 10.1007/s00464-012-2556-1

[24] Chang SK, Wang YL, Shen L (2013) Interim report: a randomized controlled trial comparing postoperative pain in single-incision laparoscopic cholecystectomy and conventional laparoscopic cholecystectomy. Asian J Endosc Surg 6: 14-20. doi:10.1111/j.1758-5910.2012.00154.x. PubMed: 22979900. 22979900 10.1111/j.1758-5910.2012.00154.x

[25] Ostlie DJ, Juang OO, Iqbal CW (2013) Single incision versus standard 4-port laparoscopic cholecystectomy: a prospective randomized trial. J Pediatr Surg 48: 209-214. doi:10.1016/j.jpedsurg.2012.10.039. PubMed: 23331817. 23331817 10.1016/j.jpedsurg.2012.10.039

[26] Pan MX, Jiang ZS, Cheng Y (2013) Single-incision vs three-port laparoscopic cholecystectomy: Prospective randomized study. World J Gastroenterol 19: 394-398. doi:10.3748/wjg.v19.i3.394. PubMed: 23372363. 23372363 10.3748/wjg.v19.i3.394 PMC3554825

[27] Sinan H, Demirbas S, Ozer MT, Sucullu I, Akyol M (2012) Single-incision laparoscopic cholecystectomy versus laparoscopic cholecystectomy:a prospective randomized study. Surg Laparosc Endosc Percutan Tech 22: 12-16. doi:10.1097/SLE.0b013e318247d9f3. PubMed: 22318052. 22318052 10.1097/SLE.0b013e3182402448

[28] Vilallonga R, Barbaros U, Sümer A, Demirel T, Fort JM et al. (2012) Single-port transumbilical laparoscopic cholecystectomy: a prospective randomised comparison of clinical results of 140 cases. J Minim Access Surg 8: 74-78. doi:10.4103/0972-9941.97586. PubMed: 22837593. 22837593 10.4103/0972-9941.97586 PMC3401720

[29] Phillips MS, Marks JM, Roberts K, Tacchino R, Onders R et al. (2012) Intermediate results of a prospective randomized controlled trial of traditional four-port laparoscopic cholecystectomy versus single-incision laparoscopic cholecystectomy. Surg Endosc 26: 1296-1303. doi:10.1007/s00464-011-2028-z. PubMed: 22083331. 22083331 10.1007/s00464-011-2028-z

[30] Noguera JF, Cuadrado A, Dolz C, Olea JM, García JC (2012) Prospective randomized clinical trial comparing laparoscopic cholecystectomy and hybrid natural orifice transluminal endoscopic surgery (NOTES) (NCT00835250). Surg Endosc 26: 3435-3441. doi:10.1007/s00464-012-2359-4. PubMed: 22648123. 22648123 10.1007/s00464-012-2359-4

[31] Sasaki A, Ogawa M, Tono C, Obara S, Hosoi N et al. (2012) Single-port versus multiport laparoscopic cholecystectomy: a prospective randomized clinical trial. Surg Laparosc Endosc Percutan Tech 22: 396-399. doi:10.1097/SLE.0b013e3182631a9a. PubMed: 23047380. 23047380 10.1097/SLE.0b013e3182631a9a

[32] Luna RA, Nogueira DB, Varela PS, Rodrigues Neto Ede O, Norton MJ et al (2013) A prospective, randomized comparison of pain, inflammatory response, and short-term outcomes between single port and laparoscopic cholecystectomy. Surg Endosc 27: 1254-1259. doi:10.1007/s00464-012-2589-5. PubMed: 23232993. 23232993 10.1007/s00464-012-2589-5

[33] Leung D, Yetasook AK, Carbray J, Butt Z, Hoeger Y et al. (2012) Single-incision surgery has higher cost with equivalent pain and quality-of-life scores compared with multiple-incision laparoscopic cholecystectomy: a prospective randomized blinded comparison. J Am Coll Surg 215: 702-708. doi:10.1016/j.jamcollsurg.2012.05.038. PubMed: 22819642. 22819642 10.1016/j.jamcollsurg.2012.05.038

[34] Zheng M, Qin M, Zhao H (2012) Laparoendoscopic single-site cholecystectomy: A randomized controlled study. Minim Invasive Ther Allied Technol 21: 113-117. doi:10.3109/13645706.2011.577787. PubMed: 21574826. 21574826 10.3109/13645706.2011.577787

[35] Marks J, Tacchino R, Roberts K, Onders R, Denoto G et al. (2011) Prospective randomized controlled trial of traditional laparoscopic cholecystectomy versus single-incision laparoscopic cholecystectomy: report of preliminary data. Am J Surg 201: 369-372. doi:10.1016/j.amjsurg.2010.09.012. PubMed: 21367381. 21367381 10.1016/j.amjsurg.2010.09.012

[36] Ma J, Cassera MA, Spaun GO, Hammill CW, Hansen PD et al. (2011) Randomized controlled trial comparing single-port laparoscopic cholecystectomy and four-port laparoscopic cholecystectomy. Ann Surg 254: 22-27. doi:10.1097/SLA.0b013e3182192f89. PubMed: 21494123. 21494123 10.1097/SLA.0b013e3182192f89

[37] Lirici MM, Califano AD, Angelini P, Corcione F (2011) Laparo-endoscopic single site cholecystectomy versus standard laparoscopic cholecystectomy: results of a pilot randomized trial. Am J Surg 202: 45-52. doi:10.1016/j.amjsurg.2010.06.019. PubMed: 21600559. 21600559 10.1016/j.amjsurg.2010.06.019

[38] Lai EC, Yang GP, Tang CN, Yih PC, Chan OC et al. (2011) Prospective randomized comparative study of single incision laparoscopic cholecystectomy versus conventional four-port laparoscopic cholecystectomy. Am J Surg 202: 254–258. doi:10.1016/j.amjsurg.2010.12.009. PubMed: 21871979. 21871979 10.1016/j.amjsurg.2010.12.009

[39] Cao ZG, Cai W, Qin MF, Zhao HZ, Yue P et al. (2011) Randomized clinical trial of single-incision versus conventional laparoscopic cholecystectomy: short-term operative outcomes. Surg Laparosc Endosc Percutan Tech 21: 311-313. doi:10.1097/SLE.0b013e31822cfacd. PubMed: 22002264. 22002264 10.1097/SLE.0b013e31822cfacd

[40] Bucher P, Pugin F, Buchs NC, Ostermann S, Morel P (2011) Randomized clinical trial of laparoendoscopic single-site versus conventional laparoscopic cholecystectomy. Br J Surg 98: 1695-1702. doi:10.1002/bjs.7689. PubMed: 21964736. 21964736 10.1002/bjs.7689

[41] Aprea G, Coppola Bottazzi E, Guida F, Masone S, Persico G (2011) Laparoendoscopic single site (LESS) versus classic video-laparoscopic cholecystectomy: a randomized prospective study. J Surg Res 66: e109-e112. PubMed: 21227454. 10.1016/j.jss.2010.11.885 21227454

[42] Tsimoyiannis EC, Tsimogiannis KE, Pappas-Gogos G, Farantos C, Benetatos N et al. (2010) Different pain scores in single transumbilical incision laparoscopic cholecystectomy versus classic laparoscopic cholecystectomy: a randomized controlled trial. Surg Endosc 24: 1842-1848. doi:10.1007/s00464-010-0887-3. PubMed: 20174950. 20174950 10.1007/s00464-010-0887-3

[43] Lee PC, Lo C, Lai PS, Chang JJ, Huang SJ et al. (2010) Randomized clinical trial of single-incision laparoscopic cholecystectomy versus minilaparoscopic cholecystectomy. Br J Surg 97: 1007-1012. doi:10.1002/bjs.7087. PubMed: 20632264. 20632264 10.1002/bjs.7087

[44] Mehmood Z, Subhan A, Ali N, Rasul S, Iqbal M et al. (2010) Four port versus single incision laparoscopic cholecystectomy. J Surg Pakistan (International) 15: 122-125.

[45] Rasić Z, Schwarz D, Nesek VA, Zoricić I, Sever M et al. (2010) Single incision laparoscopic cholecystectomy-a new advantage of gallbladder surgery. Coll Antropol 34: 595-598. PubMed: 20698134. 20698134

[46] Bresadola F, Pasqualucci A, Donini A, Chiarandini P, Anania G et al. (1999) Elective transumbilical compared with standard laparoscopic cholecystectomy. Eur J Surg 165: 29-34. doi:10.1080/110241599750007478. PubMed: 10069631. 10069631 10.1080/110241599750007478

[47] Canes D, Desai MM, Aron M, Haber GP, Goel RK et al. (2008) Transumbilical single-port surgery: evolution and current status. Eur Urol 54: 1020-1029. doi:10.1016/j.eururo.2008.07.009. PubMed: 18640774. 18640774 10.1016/j.eururo.2008.07.009

[48] Gurusamy KS, Junnarkar S, Farouk M, Davidson BR (2008) Day-case versus overnight stay for laparoscopic cholecystectomy. Cochrane Database Syst Rev 16: CD006798: CD006798 PubMed: 18677781. 10.1002/14651858.CD006798.pub2 18254116

[49] Cantore F, Boni L, Di Giuseppe M, Giavarini L, Rovera F et al. (2008) Pre-incision local inﬁltration with levobupivacaine reduces pain and analgesic consumption after laparoscopic cholecystectomy: a new device for day-case procedure. Int J Surg 6: S89–S92. doi:10.1016/j.ijsu.2008.12.033. PubMed: 19264565. 19264565 10.1016/j.ijsu.2008.12.033

[50] Kilian M, Raue W, Menenakos C, Wassersleben B, Hartmann J (2011) Transvaginal-hybrid vs. single-port-access vs. 'conventional' laparoscopic cholecystectomy: a prospective observational study. Langenbecks Arch Surg 396: 709-715. doi:10.1007/s00423-011-0769-8. PubMed: 21384187. 21384187 10.1007/s00423-011-0769-8

[51] Zahid M, Anis S, Shahid R (2011) Single incision laparoscopic cholecystectomy (SILS). Pak J Med Sci 27: 38-40.

[52] Hall TC, Dennison AR, Bilku DK, Metcalfe MS, Garcea G (2012) Single-incision laparoscopic cholecystectomy: a systematic review. Arch Surg 147: 657-666. doi:10.1001/archsurg.2012.814. PubMed: 22802063. 22802063 10.1001/archsurg.2012.814

[53] Kapischke M, Caliebe A, Tepel J, Schulz T, Hedderich J (2006) Open versus laparoscopic appendectomy: a critical review. Surg Endosc 20: 1060-1068. doi:10.1007/s00464-005-0016-x. PubMed: 16703441. 16703441 10.1007/s00464-005-0016-x

