# Supplementary material for: Single Incision versus Conventional Laparoscopic Cholecystectomy Outcomes: A Meta-Analysis of Randomized Controlled Trials
Source: PLoS One. 2013 Oct 2;8(10):e76530. doi: 10.1371/journal.pone.0076530 (PMC3788730; doi:10.1371/journal.pone.0076530)
Supplement: Table S1 — General characteristics of the 25 studies included in the meta-analysis. (DOC) [file pone.0076530.s002.doc]

**Table S1.** General characteristics of the 25 studies included in the meta-analysis.

|  | **Num** | | | **M/F ratio** | | **Age (year)** | | **BMI (** **kg/m^2^)** | | **ASA (Ⅰ/Ⅱ/Ⅲ)** | |
| --- | --- | --- | --- | --- | --- | --- | --- | --- | --- | --- | --- |
| **Study** | **SILC** | **CLC** | | **SILC** | **CLC** | **SILC** | **CLC** | **SILC** | **CLC** | **SILC** | **CLC** |
| Saad^22^,2013 | 35 | | 35 | 28/7 | 26/9 | 45 ± 17 | 49 ± 14 | 25.4 ± 2.5 | 25.4 ± 3.1 | 14/20/1 | 13/21/1 |
| Madureira^23^,2013 | 28 | | 29 | - | - | 50 ^b^ | 56 ^b^ | 27.5 ^b^ | 25.0 ^b^ | - | - |
| Chang^24^,2013 | 24 | | 26 | 10/14 | 10/16 | 49.46 ± 11.3 | 51.15 ± 12.31 | 24.13 ± 4.21 | 27.65 ± 7.79 | 10/14/0 | 9/16/0 |
| Ostlie^25^2013 | 30 | | 30 | 6/24 | 6/24 | 14.0 ± 3.2 | 13.3 ± 3.3 | - | - | - | - |
| Pan^26^,2013 | 49 | | 53 | 23/26 | 22/31 | 43.8 ± 14.0 | 45.2 ± 11.0 | 24.3 ± 6.0 | 25.1 ± 5.0 | 1.5 ± 0.2 | 1.6 ± 0.3 |
| Sinan^27^, 2012 | 17 | | 17 | 13/4 | 9/8 | 48.5 ± 8.9 | 48.7 ± 14.3 | 27.3 ± 3.1 | 27.2 ± 2.9 | - | - |
| Vilallonga^28^,2012 | 69 | | 71 | 31/38 | 35/36 | 43.2 ± 14.6 | 42.6 ± 14.6 | - | - | - | - |
| Phillips^29^,2012 | 117 | | 80 | 28/89 | 23/56 | 45.6(18 - 77) ^a^ | 44.1(19 - 68) ^a^ | 28.9(15.4-44.8)^a^ | 31.0(18.8-45)^a^ | <3^c^ | <3^c^ |
| Noguera^30^,2012 | 20 | | 20 | 0/20 | 0/20 | 49.1 ^b^ | 47.2 ^b^ | 30.8 ^b^ | 27.4 ^b^ | - | - |
| Sasaki^31^,2012 | 27 | | 27 | 14/13 | 14/13 | 56.6 ± 14.2 | 58.2 ± 12.3 | 24.4 ± 3.0 | 24.9 ± 3.4 | - | - |
| Luna^32^,2012 | 20 | | 20 | - | - | - | - | <35 ^d^ | <35 ^d^ | - | - |
| Leung^33^,2012 | 36 | | 43 | 5/31 | 16/27 | 41.8 ± 16.9 | 52.3 ± 19.8 | 28.7 ± 6.91 | 28.4 ± 6.12 | - | - |
| Zheng^34^,2012 | 30 | | 30 | 13/17 | 16/14 | 43.6 ± 11.3 | 46.8 ± 14.4 | 24.7 ± 3.4 | 25.9 ± 4.1 | - | - |
| Marks^35^, 2011 | 50 | | 33 | - | - | 40.2 ± 10.8 | 42.0 ± 14.1 | 29.4 ± 5.9 | 28.9 ± 4.6 | - | - |
| Ma ^36^, 2011 | 21 | | 22 | - | - | 57.3 ± 16.0 | 45.8 ± 11.9 | 28.2 ± 5.3 | 30.7 ± 6.1 | 2.2 ^b^ | 2.1 ^b^ |
| Lirici^37^, 2011 | 20 | | 20 | 6/14 | 6/14 | 45(26 - 63)^a^ | 50(24 - 67)^a^ | 25(18 - 29)^a^ | 27(18 - 30)^a^ | 5/14/1 | 4/12/4 |
| Lai ^38^, 2011 | 24 | | 27 | 8/16 | 11/16 | 51.7 ± 13.3 | 54.3 ± 12.0 | 25 ± 3.0 | 24.4 ±2.8 | 17/7/0 | 12/14/1 |
| Cao ^39^, 2011 | 57 | | 51 | 23/34 | 22/29 | 62.2 ± 5.1 | 59.7 ± 4.4 | 28.6 ± 4.4 | 29.1 ± 5.1 | 1.8 ± 0.5 | 1.9 ± 0.7 |
| Bucher^40^, 2011 | 75 | | 75 | - | - | 42(18-81)^a^ | 44(20-78)^a^ | 26(22 - 35)^a^ | 25(19 - 34)^a^ | 2(1 - 3)^a^ | 2(1 - 3)^a^ |
| Aprea ^41^, 2011 | 25 | | 25 | 14/16 | 6/19 | 45.5 ± 9.4 | 44.0 ± 10.0 | 25.9 ± 5.8 | 23.7 ± 4.6 | - | - |
| Tsimoyiannis^42^,2010 | 20 | | 20 | 5/15 | 1/19 | 49.2 ± 16.9 | 47.9 ± 9.8 | - | - | 1.15±0.36 | 1.20±0.41 |
| Lee ^43^, 2010 | 35 | | 35 | 13/22 | 15/20 | 51.0 ± 13.5 | 53.3 ± 15.5 | 24.2 ± 3.4 | 25.8 ± 3.0 | - | - |
| Mehamood^44^,2010 | 30 | | 30 | 2/28 | 4/26 | 44.42 ± 8.59 | 42.67 ± 9.05 | - | - | - | - |
| Rasic^45^,2010 | 48 | | 50 | 22/26 | 18/32 | 44 ± 6 | 44 ± 5.7 | 27 ± 4 | 27 ± 4 | - | - |
| Bresadola^46^,1999 | 37 | | 28 | 9/19 | 15/22 | 42 ± 20 | 45 ± 15 | - | - | <3 ^c^ | <3 ^c^ |

Data are expressed as mean ± standard deviation or as numbers.

a: Median (range); b: Mean; c: The maximum ASA; d: The maximum BMI.

BMI = body mass index; ASA = American Society of Anesthesiologists.
