# Supplementary material for: Single Incision versus Conventional Laparoscopic Cholecystectomy Outcomes: A Meta-Analysis of Randomized Controlled Trials
Source: PLoS One. 2013 Oct 2;8(10):e76530. doi: 10.1371/journal.pone.0076530 (PMC3788730; doi:10.1371/journal.pone.0076530)
Supplement: Table S2 — Operative techniques and follow-up time in the 25 studies. (DOC) [file pone.0076530.s003.doc]

**Table S2.** **Operative techniques and follow-up time in the 25 studies.**

|  | **Operative techniques** | |  |
| --- | --- | --- | --- |
| **Study** | **SILC** | **CLC** | **Follow-up time**^b^ |
| Saad^22^,2013 | A | Four ports | 12 months |
| Madureira^23^,2013 | A | Four ports | 5.92 months |
| Chang^24^,2013 | A | Four ports | 14 days |
| Ostlie^25^2013 | A | Four ports | 6 weeks |
| Pan^26^,2013 | C | Three ports | 2 months |
| Sinan^27^, 2012 | A | Four ports | E |
| Vilallonga^28^,2012 | A | Four ports | 7.3 months |
| Phillips^29^,2012 | A | Four ports | F |
| Noguera^30^,2012 | D | Three ports | 16(13-20)^a^ months |
| Sasaki^31^,2012 | A | Four ports | Postoperative results |
| Luna^32^,2012 | A | Four ports | 30 days |
| Leung^33^,2012 | - | - | G |
| Zheng^34^,2012 | A | Three ports | H |
| Marks^35^, 2011 | A | Four ports | 1 year |
| Ma ^36^, 2011 | A | Three ports | I |
| Lirici^37^, 2011 | A | Four ports | 1 month |
| Lai ^38^, 2011 | A | Four ports | 3 month |
| Cao ^39^, 2011 | B | Three ports | <30 days |
| Bucher^40^, 2011 | A | Four ports | 1 month |
| Aprea ^41^, 2011 | A | Three ports | Short period |
| Tsimoyiannis^42^,2010 | B | Four ports | - |
| Lee ^43^, 2010 | A | Four ports | Postoperative results |
| Mehamood^44^,2010 | A | Four ports | Postoperative results |
| Rasic^45^,2010 | B | Three ports | Postoperative results |
| Bresadola^46^,1999 | C | Four ports | Postoperative results |

**Operative Techniques**

A: A commercial port such as TriPort®, SILS® port was used. B: Three ports were placed through the same umbilical incision but through separate fascial incisions. C: Hybrid, suture using a straight needle was inserted. D: Endoscopic single-port.

**Follow-up time**

E: SILC 29.9 (15.7-63.3, 39.4), CLC 22.7 (8.0-56.4, 14.9); mean and standard deviation; week. F: 197 patients on the day of surgery, 194 at 1 week, 184 at 1 month, 165 at 3 months, and 56 completed the study at 12 months. G: SILC 99 days, CLC 90 days. H: SILC 9.4(4-24), CLC 11.6(8-24); median (range); month. I: SILC 19.4 ± 1.7, CLC 16.0 ± 2.4; mean and standard deviation; day.

a: median (range). b: Less than one month was considered as short period, otherwise, as a slight long period.
