# Supplementary material for: Single Incision versus Conventional Laparoscopic Cholecystectomy Outcomes: A Meta-Analysis of Randomized Controlled Trials
Source: PLoS One. 2013 Oct 2;8(10):e76530. doi: 10.1371/journal.pone.0076530 (PMC3788730; doi:10.1371/journal.pone.0076530)
Supplement: Table S3 — VASs of the 25 studies included in the meta-analysis. (DOC) [file pone.0076530.s004.doc]

**Table S3.** VASs of the 25 studies included in the meta-analysis.

|  | **VAS (3-4 hours)** | | **VAS (6-8 hours)** | | **VAS (12 hours)** | | **VAS (24 hours)** | |
| --- | --- | --- | --- | --- | --- | --- | --- | --- |
| **Study** | **SILC** | **CLC** | **SILC** | **CLC** | **SILC** | **CLC** | **SILC** | **CLC** |
| Saad^22^,2013 | - | - | - | - | - | - | 2.3 ± 1.6 | 2.1 ± 1.5 |
| Madureira^23^,2013 | 2.0 (0–7) ^a^ | 4.0 (0–10) ^a^ | - | - | - | - | 0.3 (0 - 6) ^a^ | 2.3 (0 - 10) ^a^ |
| Chang^24^,2013 | - | - | - | - | - | - | 2.3 ± 1.9 | 2.7 ± 2.6 |
| Ostlie^25^2013 | - | - | - | - | - | - | - | - |
| Pan^26^,2013 | - | - | 2.0 ± 1.5 | 3.5 ± 1.6 | - | - | - | - |
| Sinan^27^, 2012 | 5 (0 - 8) ^a^ | 5 (2 - 9) ^a^ | 2 (1-6) ^a^ | 3 (0-5) ^a^ | 2 (0 - 4) ^a^ | 2 (1 - 8) ^a^ | 1 (0 - 4) ^a^ | 1 (0 - 4) ^a^ |
| Vilallonga^28^,2012 | - | - | - | - | - | - | 2.0 ± 0.8 | 2.9 ± 1.2 |
| Phillips^29^,2012 | - | - | - | - | - | - | 4.9 | 4.4 |
| Noguera^30^,2012 | - | - | - | - | - | - | 3.03 | 4.65 |
| Sasaki^31^,2012 | - | - | - | - | - | - | 2.4 ± 1.4 | 2.6 ± 1.2 |
| Luna^32^,2012 | 3.5 ± 2.6 | 4.3 ± 2.6 | 2.1 ±1.7 | 2.8 ± 2.3 | - | - | 1.4 ± 1.6 | 0.8 ± 1.1 |
| Leung^33^,2012 | - | - | - | - | - | - | - | - |
| Zheng^34^,2012 | - | - | - | - | - | - | 2.8 ± 0.6 | 3.7 ± 1.1 |
| Marks^35^, 2011 | - | - | - | - | - | - | - | - |
| Ma ^36^, 2011 | - | - | - | - | - | - | 2.7 | 1.8 |
| Lirici^37^, 2011 | - | - | - | - | - | - | 2 (0 - 8) ^a^ | 2 (0 - 9) ^a^ |
| Lai ^38^, 2011 | - | - | 4.5(2 - 8) ^a^ | 4.0(2 - 7) ^a^ | - | - | - | - |
| Cao ^39^, 2011 | - | - | - | - | - | - | 2.3 ± 0.9 | 2.6 ±1.2 |
| Bucher^40^, 2011 | - | - | 2 (0 - 4) ^a^ | 3 (2 - 7) ^a^ | - | - | 1 (0 - 4) ^a^ | 3 (2 - 5) ^a^ |
| Aprea ^41^, 2011 | - | - | 3.9 ± 1.8 | 3.5 ± 1.6 | 4.5 ± 2.0 | 4.0 ± 1.6 | 2.8±1.3 | 2.2 ± 1.3 |
| Tsimoyiannis^42^,2010 | 0.75 ± 0.63 | 0.95± 0.75 | 1.0 ± 0.85 | 1.6 ± 0.88 | 1.65±0.67 | 1.8 ± 0.95 | 0.50 ± 0.60 | 1.55 ± 0.94 |
| Lee ^43^, 2010 | - | - | - | - | - | - | 2.1 ± 0.9 | 2.2 ± 0.8 |
| Mehamood^44^,2010 | - | - | - | - | - | - | 5.23 ± 1.52 | 2.93 ± 0.98 |
| Rasic^45^,2010 | - | - | - | - | - | - | - | - |
| Bresadola^46^,1999 | 3 (0 - 6.5) ^a^ | 3.4 (1 - 7) ^a^ | 2 (0 - 5.5) ^a^ | 3.4 (1-7.5) ^a^ | 2 (0 - 5.5) ^a^ | 3.4 (1-7.5)^a^ | 1.1(0-2.5) ^a^ | 1.1(0-3.5) ^a^ |

Data are expressed as mean ± standard deviation/mean; a: median (range). VAS = visual analogue scale/score.
