# Supplementary material for: Single Incision versus Conventional Laparoscopic Cholecystectomy Outcomes: A Meta-Analysis of Randomized Controlled Trials
Source: PLoS One. 2013 Oct 2;8(10):e76530. doi: 10.1371/journal.pone.0076530 (PMC3788730; doi:10.1371/journal.pone.0076530)
Supplement: Table S4 — Cosmetic results of the 25 studies included in the meta-analysis. (DOC) [file pone.0076530.s005.doc]

**Table S4.** Cosmetic results of the 25 studies included in the meta-analysis.

|  | **Cosmetic score** | | **The length of incision (mm)** | |
| --- | --- | --- | --- | --- |
| **Study** | **SILC** | **CLC** | **SILC** | **CLC** |
| Saad^22^,2013 | 1.65 ± 1.18^b^ | 1.57 ± 0.61^b^ | - | - |
| Madureira^23^,2013 | - | - | 40(21-58) ^a^ | 27(15-51) ^a^ |
| Chang^24^,2013 | - | - | - | - |
| Ostlie^25^2013 | - | - | - | - |
| Pan^26^,2013 | 8 ± 0.4 | 6 ± 0.2 | - | - |
| Sinan^27^, 2012 | - | - | - | - |
| Vilallonga^28^,2012 | 8.3 ± 0.9 | 6.7 ± 1.1 | - | - |
| Phillips^29^,2012 | 8.7 | 8.3 | - | - |
| Noguera^30^,2012 | - | - | - | - |
| Sasaki^31^,2012 | 9.7 ± 0.5 | 8.9 ± 1.2 | - | - |
| Luna^32^,2012 | - | - | - | - |
| Leung^33^,2012 | 8.55 | 9.24 | - | - |
| Zheng^34^,2012 | 8.9 ± 0.7 | 8.1 ± 1.5 | - | - |
| Marks^35^, 2011 | - | - | - | - |
| Ma ^36^, 2011 | 9.3 | 8.9 | - | - |
| Lirici^37^, 2011 | 83.5%(60%-100%) ^a^ | 75% (0-100%) ^a^ | 14.31 ± 4.14 | 18.8 ± 3.47 |
| Lai ^38^, 2011 | 7 (4 - 8) ^a^ | 6 (3 - 8) ^a^ | 17.6 ± 2.9 | 22.5 ± 0.5 |
| Cao ^39^, 2011 | - | - | 21.6 ± 2.4 | 30.8 ± 2.6 |
| Bucher^40^, 2011 | 6 (5 - 7) ^a, c^ | 8 (7 - 11) ^a c^ | - | - |
| Aprea ^41^, 2011 | 4.9 ± 0.2^b^ | 3.5 ± 0.5^b^ | - | - |
| Tsimoyiannis^42^,2010 | - | - | - | - |
| Lee ^43^, 2010 | 8.7 ± 1.0 | 7.7 ± 1.4 | 15.7 ± 1.0 | 20.9 ± 1.4 |
| Mehamood^44^,2010 | - | - | - | - |
| Rasic^45^,2010 | - | - | - | - |
| Bresadola^46^,1999 | - | - | - | - |

Data are expressed as mean ± standard deviation/mean; a: median (range).

b: Rated from 1 to 5; c: Scale from 5 (best) to 20.
