# Supplementary material for: Single Incision versus Conventional Laparoscopic Cholecystectomy Outcomes: A Meta-Analysis of Randomized Controlled Trials
Source: PLoS One. 2013 Oct 2;8(10):e76530. doi: 10.1371/journal.pone.0076530 (PMC3788730; doi:10.1371/journal.pone.0076530)
Supplement: Table S5 — Intraoperative outcomes of the 25 studies included in the meta-analysis. (DOC) [file pone.0076530.s006.doc]

**Table S5.** Intraoperative outcomes of the 25 studies included in the meta-analysis.

|  | **Operating time (min)** | | **Blood loss (ml)** | | **Additional instruments added** | | **Conversion to open** | |
| --- | --- | --- | --- | --- | --- | --- | --- | --- |
| **Study** | **SILC** | **CLC** | **SILC** | **CLC** | **SILC** | **CLC** | **SILC** | **CLC** |
| Saad^22^,2013 | 45.7 ± 10.9 | 35.0 ± 14.0 | - | - | 1 | 0 | 0 | 0 |
| Madureira^23^,2013 | 60.3(32 - 128)^a^ | 51.3(25 - 120)^a^ | - | - | 0 | 0 | 0 | 0 |
| Chang^24^,2013 | 76.04 | 61.12 | - | - | 0 | 0 | 0 | 0 |
| Ostlie^25^2013 | 68.6 ± 22.1 | 56.1 ± 22.1 | - | - | 0 | 0 | 0 | 0 |
| Pan^26^,2013 | 41.8 ± 17.0 | 38.5 ± 22.0 | 14 ± 6.0 | 15 ± 4.0 | 1 | 0 | 0 | 0 |
| Sinan^27^, 2012 | 124.4 ± 29.7 | 64.1 ± 26.1 | 25.9 ± 6.7 | 35.0 ± 27.0 | 0 | 0 | 0 | 0 |
| Vilallonga^28^,2012 | 63.9 ± 23.9 | 58.4 ± 20.8 | 5.8 ± 36.9 | 10 ± 35.2 | 1 | 0 | 0 | 1 |
| Phillips^29^,2012 | 57.2 | 45.2 | 14.9 | 14.2 | 1 | 0 | 0 | 0 |
| Noguera^30^,2012 | 59.80 | 47.04 | - | - | 1 | 0 | 0 | 0 |
| Sasaki^31^,2012 | 83.4 ± 18.6 | 69.4 ± 16.7 | 13.8 ± 16.8 | 17.4 ± 48.4 | 0 | 0 | 0 | 0 |
| Luna^32^,2012 | 92 ± 27.7 | 41.9 ± 14 | - | - | 4 | 0 | 0 | 0 |
| Leung^33^,2012 | 72.9 | 46.2 | - | - | 5 | 0 | 0 | 0 |
| Zheng^34^,2012 | 55.6 ± 25.7 | 42.7 ± 18.6 | - | - | 2 | 0 | 0 | 0 |
| Marks^35^, 2011 | 58.2 ± 25.3 | 44.0 ± 16.2 | 27.6 ± 102.1 | 10.4 ± 14.9 | 1 | 0 | 0 | 0 |
| Ma ^36^, 2011 | 88.5 | 44.8 | - | - | 14^c^ | 0 | 0 | 0 |
| Lirici^37^, 2011 | 75 (45 - 115)^a^ | 35 (15 - 280)^a^ | - | - | 2 | 0 | 0 | 1 |
| Lai ^38^, 2011 | 46.5 ± 20.1 | 43.5 ± 15.4 | 1 (1 - 30)^a^ | 1 (1 - 10)^a^ | 0 | 0 | 0 | 0 |
| Cao ^39^, 2011 | 55.2 ± 12.4 | 46.3 ± 10.8 | 14 ± 4.5 | 12 ± 3.8 | 1 | 0 | 1 | 0 |
| Bucher^40^, 2011 | 66 (32 - 109)^a^ | 64 (38 - 117)^a^ | - | - | 2 | 0 | 0 | 0 |
| Aprea ^41^, 2011 | 41.3 ± 12.0 | 35.6 ± 5.8 | - | - | 2 | 1 | 0 | 0 |
| Tsimoyiannis^42^,2010 | 49.65 ± 9.02 | 37.3 ± 9.16 | 9.9 ± 14.38 | 8.50 ± 6.30 | - | - | - | - |
| Lee ^43^, 2010 | 71.7 ± 11.6 | 48.4 ± 10.5 | - | - | 2 | 1 | 0 | 0 |
| Mehamood^44^,2010 | 80.17 ± 30.16 | 38.50 ± 8.92 | - | - | - | - | - | - |
| Rasic^45^,2010 | 46 ± 3.5 | 43 ± 4 | - | - | 1 | 0 | 0 | 0 |
| Bresadola^46^,1999 | 94 ± 29 | 85 ± 33 | - | - | 17^b^ | 0 ^b^ | 0 ^b^ | 0 ^b^ |

Data are expressed as mean ± standard deviation/mean in operating time and blood loss, or as numbers in conversion number.

a: Median (range); b: SILC 41, CLC 37; c: Initial 11 cases included.
