# Supplementary material for: Single Incision versus Conventional Laparoscopic Cholecystectomy Outcomes: A Meta-Analysis of Randomized Controlled Trials
Source: PLoS One. 2013 Oct 2;8(10):e76530. doi: 10.1371/journal.pone.0076530 (PMC3788730; doi:10.1371/journal.pone.0076530)
Supplement: Table S6 — Postoperative complications of the 25 studies included in the meta-analysis. (DOC) [file pone.0076530.s007.doc]

**Table S6.** Postoperative complications of the 25 studies included in the meta-analysis.

|  | **Incisional hernia** | | **Wound complications** | | | **Retained stones** | | **Bile duct injury** | | **Bile leakage** | | | **Bleeding** | | **Overall morbidity** | |
| --- | --- | --- | --- | --- | --- | --- | --- | --- | --- | --- | --- | --- | --- | --- | --- | --- |
| **Study** | **SILC** | **CLC** | **SILC** | | **CLC** | **SILC** | **CLC** | **SILC** | **CLC** | **SILC** | | **CLC** | **SILC** | **CLC** | **SILC** | **CLC** |
| Saad^22^,2013 | 1 | 0 | 5 | 0 | | 1 | 0 | 0 | 0 | 0 | 0 | | 0 | 0 | 8 | 0 |
| Madureira^23^,2013 | 0 | 0 | 10 | 14 | | 0 | 0 | 0 | 0 | 0 | 0 | | 0 | 0 | 10 | 14 |
| Chang^24^,2013 | 0 | 0 | 1 | 0 | | 0 | 0 | 0 | 0 | 0 | 0 | | 0 | 0 | 1 | 0 |
| Ostlie^25^2013 | 0 | 0 | 0 | 0 | | 0 | 0 | 0 | 0 | 0 | 0 | | 0 | 0 | 0 | 0 |
| Pan^26^,2013 | 0 | 0 | 7 | 5 | | 0 | 0 | 0 | 0 | 0 | 0 | | 0 | 0 | 7 | 5 |
| Sinan^27^, 2012 | 1 | 0 | 0 | 0 | | 0 | 0 | 0 | 0 | 0 | 0 | | 0 | 0 | 1 | 1 |
| Vilallonga^28^,2012 | 1 | 2 | 2 | 3 | | 0 | 0 | 0 | 0 | 0 | 0 | | 0 | 0 | 3 | 5 |
| Phillips^29^,2012 | 4 | 1 | 12 | 2 | | 1 | 1 | 0 | 0 | 0 | 0 | | 0 | 0 | 45 | 27 |
| Noguera^30^,2012 | 0 | 0 | 1 | 1 | | 0 | 0 | 0 | 0 | 0 | 0 | | 0 | 1 | 1 | 2 |
| Sasaki^31^,2012 | 0 | 0 | 0 | 0 | | 1 | 0 | 0 | 0 | 1 | 2 | | 0 | 0 | 2 | 3 |
| Luna^32^,2012 | 0 | 0 | 1 | 2 | | 0 | 0 | 0 | 0 | 0 | 0 | | 0 | 0 | 1 | 2 |
| Leung^33^,2012 | 0 | 0 | 0 | 0 | | 0 | 0 | 0 | 0 | 0 | 0 | | 0 | 0 | 0 | 0 |
| Zheng^34^,2012 | 0 | 0 | 0 | 0 | | 0 | 0 | 0 | 0 | 0 | 0 | | 0 | 0 | 2 | 1 |
| Marks^35^, 2011 | 1 | 0 | 0 | 0 | | 0 | 0 | 0 | 0 | 0 | 0 | | 0 | 0 | 13 | 7 |
| Ma ^36^, 2011 | 1 | 0 | 4 | 4 | | 1 | 0 | 0 | 0 | 0 | 0 | | 0 | 0 | 6 | 4 |
| Lirici^37^, 2011 | 0 | 0 | 0 | 0 | | 0 | 0 | 0 | 1 | 0 | 0 | | 1 | 1 | 1 | 2 |
| Lai ^38^, 2011 | 0 | 0 | 0 | 1 | | 0 | 0 | 0 | 0 | 0 | 0 | | 0 | 0 | 0 | 1 |
| Cao ^39^, 2011 | 0 | 0 | 1 | 1 | | 1 | 0 | 0 | 0 | 1 | 0 | | 0 | 0 | 2 | 1 |
| Bucher^40^, 2011 | 0 | 0 | 3 | 4 | | 0 | 0 | 0 | 0 | 0 | 0 | | 0 | 0 | 3 | 4 |
| Aprea ^41^, 2011 | 0 | 0 | 0 | 0 | | 0 | 0 | 0 | 0 | 0 | 0 | | 0 | 0 | 0 | 0 |
| Tsimoyiannis^42^,2010 | 0 | 0 | 0 | 0 | | 0 | 0 | 2 | 1 | 0 | 0 | | 0 | 0 | 2 | 1 |
| Lee ^43^, 2010 | 0 | 0 | 0 | 0 | | 0 | 0 | 0 | 0 | 6 | 4 | | 0 | 0 | 7 | 5 |
| Mehamood^44^,2010 | 0 | 0 | 2 | 0 | | 0 | 0 | 0 | 0 | 0 | 0 | | 0 | 0 | 2 | 0 |
| Rasic^45^,2010 | 0 | 0 | 0 | 0 | | 0 | 0 | 0 | 0 | 0 | 0 | | 0 | 0 | 0 | 0 |
| Bresadola^46^,1999 | 0 | 0 | 0 | 0 | | 0 | 0 | 0 | 0 | 0 | 0 | | 0 | 0 | 0 | 0 |
