# Supplementary material for: Single Incision versus Conventional Laparoscopic Cholecystectomy Outcomes: A Meta-Analysis of Randomized Controlled Trials
Source: PLoS One. 2013 Oct 2;8(10):e76530. doi: 10.1371/journal.pone.0076530 (PMC3788730; doi:10.1371/journal.pone.0076530)
Supplement: Table S7 — Recovery outcomes of the 25 studies included in the meta-analysis. (DOC) [file pone.0076530.s008.doc]

**Table S7.** Recovery outcomes of the 25 studies included in the meta-analysis.

|  | **Hospital stay (d)** | | **Initial oral intake (h)** | | | **Time to resume work(d)** | |
| --- | --- | --- | --- | --- | --- | --- | --- |
| **Study** | **SILC** | **CLC** | **SILC** | **CLC** | | **SILC** | **CLC** |
| Saad^22^,2013 | 3.1 ± 0.6 | 3.0 ± 0.2 | - | | - | - | - |
| Madureira^23^,2013 | - | - | - | | - | - | - |
| Chang^24^,2013 | - | - | - | | - | - | - |
| Ostlie^25^2013 | 1.01 ± 0.54 | 0.90 ± 0.12 | 3.8 ± 4.7 | | 5.2 ± 9.2 | 6.1 ± 3.1 | 6.0 ± 4.7 |
| Pan^26^,2013 | 1.0 ± 0.5 | 1.0 ± 0.2 | - | | - | - | - |
| Sinan^27^, 2012 | - | - | - | | - | - | - |
| Vilallonga^28^,2012 | 1.6 ± 0.9 | 1.0 ± 0.3 | 10.4 ± 5.1 | | 10.7 ± 5.9 | - | - |
| Phillips^29^,2012 | - | - | - | | - | - | - |
| Noguera^30^,2012 | - | - | - | | - | - | - |
| Sasaki^31^,2012 | 3.4 ± 0.7 | 3.4 ± 0.6 | 24.0 ± 2.4 | | 24.0 ± 2.4 | - | - |
| Luna^32^,2012 | - | - | - | | - | - | - |
| Leung^33^,2012 | 1.13 | 0.83 | - | | - | 12.7 | 9.0 |
| Zheng^34^,2012 | 3.7 ± 1.3 | 3.8 ± 0.8 | - | | - | - | - |
| Marks^35^, 2011 | - | - | - | | - | - | - |
| Ma ^36^, 2011 | - | - | - | | - | - | - |
| Lirici^37^, 2011 | 2.5 (2 - 7)^a^ | 2.65 (2 - 9)^a^ | - | | - | - | - |
| Lai ^38^, 2011 | 1.5 ± 0.6 | 1.8 ± 1.2 | - | | - | - | - |
| Cao ^39^, 2011 | 2.1 ± 1.1 | 2.8 ± 0.8 | - | | - | 5.6 ± 1.6 | 5.0 ± 1.6 |
| Bucher^40^, 2011 | 0 (0 - 2)^a^ | 1(0 - 5)^a^ | - | | - | 10 (5 - 14)^a^ | 12(11-15)^a^ |
| Aprea ^41^, 2011 | 1.2 ± 0.4 | 1.16 ± 0.37 | - | | - | - | - |
| Tsimoyiannis^42^,2010 | 1.25 ± 0.44 | 1.10 ± 0.44 | - | | - | - | - |
| Lee ^43^, 2010 | 2.4 ± 0.8 | 2.9 ± 0.4 | - | | - | 5.3 ± 2.0 | 5.9 ± 2.3 |
| Mehamood^44^,2010 | 1.70 ± 0.79 | 1.00 ± 0.00 | - | | - | - | - |
| Rasic^45^,2010 | 2 ± 0.6 | 2 ± 0.5 | - | | - | - | - |
| Bresadola^46^,1999 | 3 (2 - 4)^a^ | 3 (2 - 4)^a^ | - | | - | - | - |

Data are expressed as mean ± standard deviation/mean; a: Median (range).
